# Cutaneous adverse events due to checkpoint inhibitors – a retrospective analysis at a tertiary referral hospital in Switzerland 2019-2022

**DOI:** 10.3389/fonc.2024.1485594

**Published:** 2024-12-05

**Authors:** Clara Furrer-Matcau, Chloé Sieber, Dirk Lehnick, Christoph Urs Brand, Balthasar Hug

**Affiliations:** ^1^ Dermatology and Allergology, Cantonal Hospital Lucerne, Lucerne, Switzerland; ^2^ Biostatistics and Methodology, Clinical Trials Unit Central Switzerland, Lucerne, Switzerland; ^3^ Faculty of Health Sciences and Medicine, University of Lucerne, Lucerne, Switzerland; ^4^ Department of Medicine, Cantonal Hospital Lucerne, Lucerne, Switzerland; ^5^ Community Medicine, Faculty of Health Sciences and Medicine, University of Lucerne, Lucerne, Switzerland

**Keywords:** immune-related cutaneous adverse events, checkpoint inhibitor therapy, immunotherapy, dermatological side-effect, skin, cancer

## Abstract

**Introduction:**

Checkpoint inhibitors are increasingly important in anti-cancer treatment. Therefore, knowledge of immune-related cutaneous adverse events (ir-cAE) is crucial for therapy management and continuation.

**Objective:**

The study aimed to analyze the incidence of cutaneous adverse events caused by checkpoint inhibitor therapy, including their clinical presentation, management, and impact on further treatment.

**Methods:**

This is a descriptive, monocentric retrospective study that uses data from the electronic health record system at a tertiary referral hospital in Central Switzerland from September 2019 to September 2022. The electronic health records of patients who received a therapy with checkpoint inhibitors were examined for age, sex, type of immunotherapy, time to occurrence of ir-cAEs, characteristics of the ir-cAEs, the treatment approach, and the continuation or cessation of the therapy due to ir-cAEs.

**Results:**

Out of 431 patients, for 131 patients (30.4%) at least one ir-cAE event was documented. In particular, 109 (25.3%) experienced pruritus and 61 (14.2%) showed a maculopapular exanthema. The severity of the ir-cAE was mild in 88 patients (67.2% out of those with ir-cAEs). Ir-cAE were observed in 10 out of 20 patients (50%) treated with ipilimumab/nivolumab and in 15 out of 24 (62.5%) treated with durvalumab. In 15 patients (3.5%), checkpoint inhibitor therapy had to be discontinued due to cutaneous side effects.

**Conclusions:**

This study showed that approximately one third of the patients experienced ir-cAEs. The most frequently observed ir-cAEs were pruritus, maculopapular exanthema and xerosis cutis. In general, the dermatological manifestations are mild and responsive to topical treatment or self-limiting with no requirement for treatment interruption.

## 1 Introduction

In 2011, the introduction of the first immune checkpoint inhibitor (CPI) ipilimumab, a cytotoxic T-lymphocyte-associated protein 4 (CTLA-4) antibody, fundamentally changed cancer treatment for metastatic or unresectable melanoma (1–3).

Since then, CPIs have revolutionized cancer therapy with the introduction of 6 additional CPI (pembrolizumab, nivolumab, cemiplimab, atezolizumab, durvalumab, and avelumab) and have become indispensable in today’s cancer treatment (4–9). Specifically, for multiple tumor types such as non-small-cell lung cancer (NSCLC), head and neck tumors, or gynecological tumors, CPIs have become an important aspect in the treatment (10–12). Further CPIs are currently under development.

In general, CPIs are small monoclonal antibodies that target negative immune checkpoints (like the first CPI ipilimumab to the CTLA-4 receptor/pathway) or the programmed cell death-1 PD-1 (pembrolizumab, nivolumab and cemiplimab) and programmed cell death-ligand 1 PD-L1 (avelumab, atezolizumab and durvalumab) (13, 14). A complex network of costimulatory and inhibitory signals coordinates these pathways (15). The targeted receptors are predominantly presented on the surface of T-lymphocytes and play a crucial role in balancing the immune system and maintaining immune tolerance through their inhibitory functions (6, 16). However, tumor cells also have inhibitory immune checkpoints on their surface that the immune system recognizes as endogenous, allowing them to escape elimination in the context of immune evasion (6, 17, 18). The CTLA-4 receptors are essential for the early immune response in the lymph node. They regulate and control the activation of T-cells through their inhibitory function (17, 18). The PD-1 and PD-L1 pathways are more active in the periphery during a later phase of the immune response. They regulate and perpetuate the immune system by counteracting autoimmunity (17, 19).

The removal of the negative regulation from the immune system leads to a non-specific activation of the entire immune system. Thus, immune-related adverse events (irAE) may affect virtually any organ of the body and occur in more than 60% of patients receiving immunotherapy (16, 20, 21). According to the literature, colitis, hypophysitis, and rash are more commonly associated with anti-CTLA-4 therapy, while pneumonitis, thyroiditis, and arthralgia are more commonly associated with anti-PD-1/PD-L1 therapy (6, 17, 22, 23). Many studies show that patients undergoing therapy with anti-CTLA-4 show an increased risk of developing irAEs and more severe grades of irAEs (according to the CTCAE criteria) than patients treated with anti-PD-1/Anti-PD-L1 (3, 6, 10, 24–26). Some studies hypothesize that the timing and location of pathways in the immune system cascade may explain the occurrence and severity of side effects (17).

Skin eruptions are one of the first irAEs to appear during CPI therapy (6, 27–30). The most common immune-related cutaneous adverse events (ir-cAE) described in the literature are maculopapular rash, pruritus, lichenoid dermatitis, eczematous reaction, bullous pemphigoid, vitiligo and psoriasis (20, 27). In addition, it is important to note that CPI therapy can lead to serious adverse events, including Stevens-Johnson syndrome/toxic epidermal necrolysis (SJS/TEN), drug rash with eosinophilia and systemic symptoms (DRESS), and acute generalized exanthematous pustulosis (AGEP) (31, 32). These adverse effects should be carefully monitored and reported.

Given the growing use of CPI in routine medical practice for treating various tumors, it is crucial to have an understanding of the potential adverse events, particularly cutaneous side effects, which may manifest first, and their management (7, 27). However, only a limited number of larger studies have investigated ir-cAEs caused by CPI, with some conducted in Switzerland. The primary aim of our study was to analyze the incidence of ir-cAEs under CPI treatment, including their clinical presentation, management, and impact on further immunotherapy treatment. The second aim of this study was to descriptively explore the relationship between specific patient and tumor characteristics and ir-cAEs.

## 2 Materials and methods

We performed a retrospective, monocentric descriptive study based on patient data collected between 09/2019 and 09/2022 at the Lucerne Cantonal Hospital extracted from electronic health records. The Lucerne Cantonal Hospital is part of the LUKS Group, a tertiary referral hospital in Central Switzerland. It features more than 8000 employees, over 48500 inpatients and 920 000 ambulatory patient contacts per year. As the largest hospital in Central Switzerland, all diseases except organ transplantations are covered.

An ethics application was filed and approved by the Ethics committee of Northwestern and Central Switzerland (Nr. 2022-01848).

### 2.1 Participants

**Figure 1:**
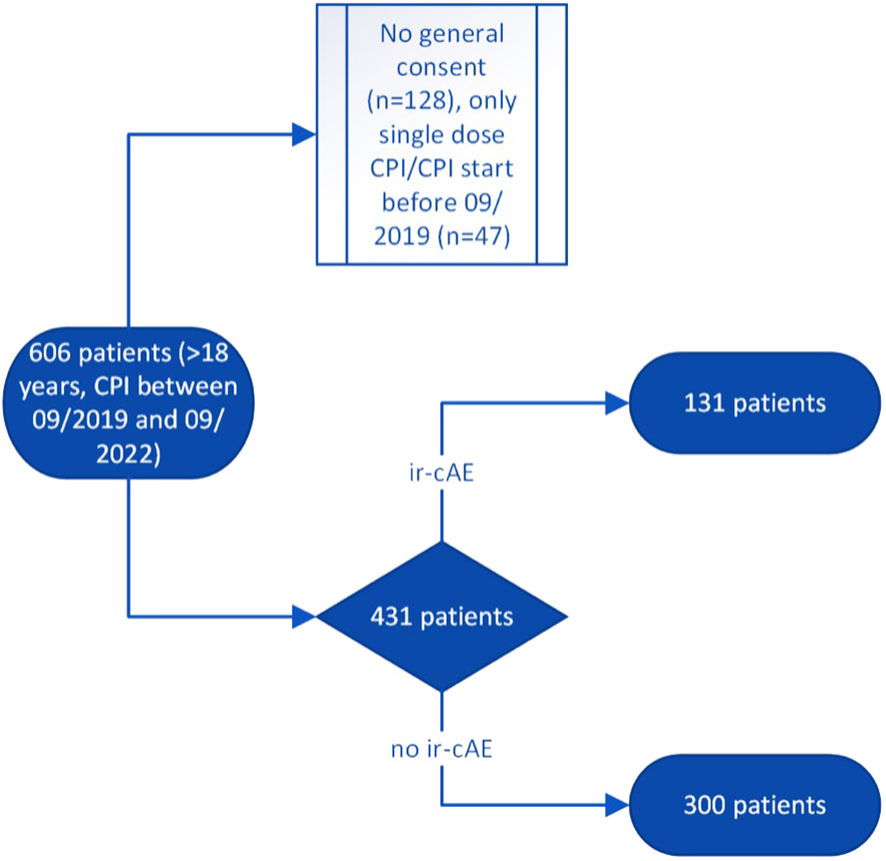
Flow chart with inclusion and exclusion criteria. ir-cAE, immune-related cutaneous adverse event; CPI, Checkpoint inhibitor therapy.

Included were patients who were at least 18 years old, had a signed general consent and underwent currently or in the past an immunotherapy with pembrolizumab, nivolumab, ipilimumab, avelumab, durvalumab or atezolizumab. Exclusion criteria included initiation of therapy prior to 2019, patients receiving a single dose, and other causes of skin lesions (see **Figure 1**).

### 2.2 Materials

Following patient data was derived from the electronic health record: age, sex, type of immunotherapy, time to occurrence of ir-cAEs, basic characteristics of the ir-cAEs (i.e., maculopapular exanthema, pruritus, xerosis cutis), whether the patient was seen by a dermatologist, how they were treated, and whether CPI therapy had to be stopped or paused due to ir-cAEs. Ir-cAEs and the type of tumor being treated were further completed by manual search in the notes’ free-text. As described in the literature, we divided the ir-cAEs into maculopapular exanthema, pruritus, xerosis cutis, eczematous reaction, lichenoid dermatitis, bullous exanthema, psoriasis, other skin diseases like vitiligo or effluvium and severe skin eruptions like SJS/TEN and DRESS (20, 27). If a cutaneous lesion was documented as “exanthema” without any further description, it was summarized as “maculopapular exanthema”. The severity classification of ir-cAEs according to the Common Terminology Criteria of Adverse Events (CTCAE) was not possible to assess retrospectively. Therefore, the severity classification was based on the clinical presentation, the treatment and whether it was possible to continue the immunotherapy or not. The ir-cAEs were classified into three categories: mild (no treatment/topical treatment + continuation of therapy), moderate (systemic treatment with or without topical treatment + continuation of therapy), and severe (therapy stopped due to ir-cAEs). The different tumor types treated with immunotherapy were grouped according to the ICD-10 classification, 10. Revision, German Modification, Version 2024 (Malignant neoplasms C00-C97).

### 2.3 Statistical analysis

Descriptive statistics characterizing study participants, their primary cancer, their CPI therapies, ir-cAEs, and the type of treatment for ir-cAEs are presented in various tables and cross-tables. Categorical information is described by frequency counts and percentages and continuous information by medians and interquartile range (IQR). Categorical information related to CPI therapy as well as to the treatment of ir-cAEs are also visualized in bar plots. The analyses were conducted in R, version 4.3.1 using the RStudio environment, version 2023.6.0.421 (33, 34).

## 3 Results

A total of 606 patients received immunotherapy within 3 years, of which 431 patients were evaluable. 128 patients had no signed general consent and were excluded. For the remaining 47 patients, therapy was started before the start of the evaluation in September 2019 or they received only a single dose within the 3 years at Lucerne Cantonal Hospital because of external oncological care. Accordingly, no evaluation was possible (see **Figure 1**).

### 3.1 Baseline characteristics of patients with ir-cAE

The 431 patients had a median age of 67 years (IQR 58-74; **Table 1**). Of all patients, 279 (64.7%) were male and 152 (35.3%) were female. The most frequent tumor types were malignant neoplasms of the respiratory and intrathoracic organs (C30-39) (n=186/431, 43.2%), melanoma and other malignant neoplasms of the skin (C43-44) (n=75/431, 17.4%) and malignant neoplasms of the digestive organs (C15-26) (n=53/431, 12.3%). 210 of the 431 patients were treated with pembrolizumab (48.7%), 91 patients with atezolizumab (21.1%), 81 patients with nivolumab (18.8%), 24 patients received durvalumab (5.6%) and 5 patients were treated with avelumab (1.2%). 20 patients (4.6%) received ipilimumab and nivolumab as combination therapy.131 of the 431 patients developed ir-cAEs (30.4%). In total, 241 skin toxicities were documented. 20.0% of all patients (n=86/431) developed more than one ir-cAE due to therapy with CPI.

**Table 1:** Baseline characteristics of patients.

	Total (N=431)
Age [years]	
Median	67.0
IQR (Q1 - Q3)	16.0 (58.0 - 74.0)
Min - max	21.0 - 93.0
Sex, n (%)	
Male	279 (64.7%)
Female	152 (35.3%)
Primary cancer, n (%)	
Malignant neoplasms of lip, oral cavity and pharynx (C00-14)	24 (5.6%)
Malignant neoplasms of digestive organs (C15-26)	53 (12.3%)
Malignant neoplasms of respiratory and intrathoracic organs (C30-39)	186 (43.2%)
Melanoma and other malignant neoplasms of skin (C43-44)	75 (17.4%)
Malignant neoplasm of breast and female genital organs (C50-C63)	27 (6.3%)
Malignant neoplasms of urinary tract (C64-68)	46 (10.7%)
Other malignant neoplasm (<10 patients) ^a^	20 (4.6%)
Immunotherapy, n (%)	
Anti-PD-L1	
Atezolizumab	91 (21.1%)
Avelumab	5 (1.2%)
Durvalumab	24 (5.6%)
Combined therapy (CTLA-4 and Anti-PD-1)	
Ipilimumab & Nivolumab	20 (4.6%)
Anti-PD-1	
Nivolumab	81 (18.8%)
Pembrolizumab	210 (48.7%)
Number of immune checkpoint-induced cutaneous adverse events per patient, n (%)	
0	300 (69.6%)
1	45 (10.4%)
2	65 (15.1%)
3	18 (4.2%)
4	3 (0.7%)

a Includes 7 patients with sarcoma, 5 patients with neuroendocrine carcinoma, 4 patients with Hodgkin´s lymphoma, 4 patients with prostate carcinoma.

In our study, 62.5% of the patients who received durvalumab (n=15/24) developed ir-cAEs, compared to 50% of the patients who received nivolumab and ipilimumab (n=10/20) followed by pembrolizumab (32.4%, n=68/210), nivolumab (23.5%, n=19/81), avelumab (20%, n=1/5) and atezolizumab (19.8%, n=18/91; see **Table 2**). Of the patients with melanoma and malignant neoplasm of the skin, 41.3% (n=31/75) developed ir-cAEs. Among patients with malignant neoplasms of the respiratory and intrathoracic organs, 34.4% (n=64/186) reported ir-cAEs, and for those with malignant neoplasms of the lip, oral cavity and pharynx 33.3% (n=8/24). The median age of patients presenting ir-cAE was 68 years (IQR 60-75) versus 67 years (IQR 58-74) for patients without reported ir-cAE. 30.5% of them were men (n=85/279) and 30.3% women (n=46/152) treated with CPI developed ir-cAEs. The median time to occurrence of ir-cAEs was 54 days (IQR 21-132).

**Table 2:** Number and percentages of patients for whom at least one cutaneous adverse event (ir-cAE) due to CPI was documented.

Immune checkpoint induced cutaneous adverse events	Any ir-cAE		Total
	Yes	No	
Patients, N (%)	131 (30.4)	300 (69.6)	431(100)
Immunotherapy, n (%)			
Anti-PD-L1			
Atezolizumab	18 (19.8)	73 (80.2)	91 (100)
Avelumab	1 (20.0)	4 (80.0)	5 (100)
Durvalumab	15 (62.5)	9 (37.5)	24 (100)
Combined therapy (CTLA-4 and Anti-PD-1)			
Ipilimumab &Nivolumab	10 (50.0)	10 (50.0)	20 (100)
Anti-PD-1			
Nivolumab	19 (23.5)	62 (76.5)	81 (100)
Pembrolizumab	68 (32.4)	142 (67.6)	210 (100)
Primary cancer, n (%)			
Malignant neoplasms of lip, oral cavity and pharynx (C00-14)	8 (33.3)	16 (66.7)	24 (100)
Malignant neoplasms of digestive organs (C15-26)	10 (18.9)	43 (81.1)	53 (100)
Malignant neoplasms of respiratory and intrathoracic organs (C30-39)	64 (34.4)	122 (65.6)	186 (100)
Melanoma and other malignant neoplasms of skin (C43-44)	31 (41.3)	44 (58.7)	75 (100)
Malignant neoplasm of breast and female genital organs (C50-C63)	5 (18.5)	22 (81.5)	27 (100)
Malignant neoplasms of urinary tract (C64-68)	12 (26.1)	34 (73.9)	46 (100)
Other malignant neoplasm (<10 patients) ^a^	1 (5.0)	19 (95.0)	20 (100)
Age [years]			
Median (IQR)	68 (60-75)	67 (58-74)	
Min - max	27 - 92	21 - 93	
Sex, n (%)			
Male	85 (30.5)	194 (69.5)	279
Female	46 (30.3)	106 (69.7)	152

a Includes 7 patients with sarcoma, 5 patients with neuroendocrine carcinoma, 4 patients with Hodgkin´s lymphoma, 4 patients with prostate carcinoma.

### 3.2 Characteristics of ir-cAE

**Table 3** describes the clinical and demographic characteristics of the patients suffering from one of the most frequently reported ir-cAEs. Out of the 431 patients treated with CPI, 109 developed pruritus (25.3%), 61 patients had a maculopapular exanthema (14.2%), 36 experienced xerosis cutis (8.4%), 24 reported other cutaneous adverse events like vitiligo (5.6%) and 9 had an eczematous reaction (2.1%). Bullous and lichenoid reaction were each seen once. No life-threatening events were documented within the observation period of three years.

**Table 3:** Number and percentages of patients for whom at least one cutaneous adverse event due to CPI was documented by ir-cAE.

Categories	Pruritus	Maculopapular exanthema	Xerosis cutis	Other skin toxicities^a^	Eczema	Bullous pemphigoid	Lichenoid	Total number of patients per category (row)
Overall	109 (25.3)	61 (14.2)	36 (8.4)	24 (5.6)	9 (2.1)	1 (0.2)	1 (0.2)	431
Immunotherapy								
Anti-PD-L1								
Atezolizumab	16 (17.6)	7 (7.7)	8 (8.8)	3 (3.3)	1 (1.1)	0 (0.0)	0 (0.0)	91 (21.1)
Avelumab	1 (20.0)	1 (20.0)	0 (0.0)	0 (0.0)	0 (0.0)	0 (0.0)	0 (0.0)	5 (1.2)
Durvalumab	13 (54.2)	4 (16.7)	5 (20.8)	2 (8.3)	1 (4.2)	0 (0.0)	0 (0.0)	24 (5.6)
Combined therapy (CTLA-4 and Anti-PD-1)								
Ipilimumab & Nivolumab	9 (45.0)	6 (30.0)	0 (0.0)	2 (10.0)	0 (0.0)	0 (0.0)	0 (0.0)	20 (4.6)
Anti-PD-1								
Nivolumab	17 (21.0)	12 (14.8)	1 (1.2)	5 (6.2)	0 (0.0)	0 (0.0)	0 (0.0)	81 (18.8)
Pembrolizumab	53 (25.2)	31 (14.8)	22 (10.5)	12 (5.7)	7 (3.3)	1 (0.5)	1 (0.5)	210 (48.7)
Primary cancer								
Malignant neoplasms of lip, oral cavity and pharynx (C00-14)	8 (33.3)	2 (8.3)	2 (8.3)	1 (4.2)	1 (4.2)	1 (4.2)	0 (0.0)	24 (5.6)
Malignant neoplasms of digestive organs (C15-26)	10 (18.9)	4 (7.5)	5 (9.4)	0 (0.0)	0 (0.0)	0 (0.0)	0 (0.0)	53 (12.3)
Malignant neoplasms of respiratory and intrathoracic organs (C30-39)	54 (29.0)	29 (15.6)	21 (11.3)	8 (4.3)	5 (2.7)	0 (0.0)	0 (0.0)	186 (43.2)
Melanoma and other malignant neoplasms of skin (C43-44)	23 (30.7)	17 (22.7)	5 (6.7)	13 (17.3)	3 (4.0)	0 (0.0)	0 (0.0)	75 (17.4)
Malignant neoplasm of breast and female genital organs (C50-C63)	3 (11.1)	3 (11.1)	1 (3.7)	0 (0.0)	0 (0.0)	0 (0.0)	0 (0.0)	27 (6.3)
Malignant neoplasms of urinary tract (C64-68)	10 (21.7)	5 (10.9)	2 (4.3)	2 (4.3)	0 (0.0)	0 (0.0)	1 (2.2)	46 (10.7)
Other malignant neoplasm (<10 patients) ^b^	1 (5.0)	1 (5.0)	0 (0.0)	0 (0.0)	0 (0.0)	0 (0.0)	0 (0.0)	20 (4.6)
Age (Years)								
Median (IQR)	68 (59-75)	67 (60-76)	66 (60-74)	70 (54-76)	70 (58-77)	83 (83-83)	84 (84-84)	_
Min - max	38 - 91	38 - 91	46 - 92	27 - 84	50 - 86	83 - 83	84 - 84	_
Sex								
Male	73 (26.2)	39 (14.0)	20 (7.2)	11 (3.9)	5 (1.8)	0 (0.0)	1 (0.4)	279 (64.7)
Female	36 (23.7)	22 (14.5)	16 (10.5)	13 (8.6)	4 (2.6)	1 (0.7)	0 (0.0)	152 (35.3)
Time to ir-cAE ^c^								
Median (IQR)	53 (17-117)	35 (18-91)	72 (31-148)	42 (25-194)	53 (21-147)	29 (29-29)	279 (279-279)	
Min - max	0 - 776	2 - 379	0 - 441	5 - 544	5 - 342	29 - 29	279 - 279	

a Other skin toxicities include bullous exanthema, lichenoid exanthema, vitiligo and effluvium.

b Includes 7 patients with sarcoma, 5 patients with neuroendocrine carcinoma, 4 patients with Hodgkin´s lymphoma, 4 patients with prostate carcinoma.

c Ir-cAE=immune-related cutaneous adverse events.

Pruritus occurred as the ir-cAE in 54.2% (n=13/24) of patients treated with durvalumab, 45.0% (n=9/20) of patients treated with ipilimumab and nivolumab, and 25.2% (n=53/210) of patients treated with pembrolizumab. The therapy resulting in the highest proportion of maculopapular exanthema was due to ipilimumab and nivolumab (n=6/20, 30.0%), whereas the highest proportion of xerosis cutis was observed under durvalumab (n=5/24, 20.8%).

Pruritus was most frequently present in patients with malignant neoplasms of the lip, oral cavity and pharynx (n=8/24, 33.3%) and in patients with melanoma and other malignant neoplasm of skin (n=23/75, 30.7%). 22.7% of the patients with ir-cAEs and suffering from melanoma and other malignant neoplasm of skin reported maculopapular exanthema (n=17/75), whereas 17.3% of this subgroup reported other skin toxicities (n=13/75). Xerosis cutis was observed in 11.3% (n=21/186) of the patients with malignant neoplasms of respiratory and intrathoracic organs.

No remarkable differences in age and gender were identified among the various skin lesions.

Maculopapular exanthema developed at a median time of 35 days (IQR 18-91), whereas xerosis cutis occurred at a median time of 72 days (IQR 31-148). Pruritus and eczema at a median time of 53 days (IQR 17-117, IQR 21-147). The shortest times to onset of ir-cAEs at the individual level were 0 days for pruritus and xerosis cutis, whereas the longest time to onset of ir-cAE was 776 days for pruritus.

Twenty-four patients of the 131 patients with ir-cAE (18.3%) underwent dermatological examination. Of those, 87.5% showed pruritus (n=21/24).

88 patients of the 131 patients with ir-cAE (67.2%) required either no treatment or only topical therapy owing to the mild ir-cAE, 28 out of 131 patients (21.4%) received systemic treatment for ir-cAE with a moderate severity and 15 patients (11.5%) had to discontinue immunotherapy due to severe ir-cAE.

### 3.3 Treatment decisions

**Figure 2:**
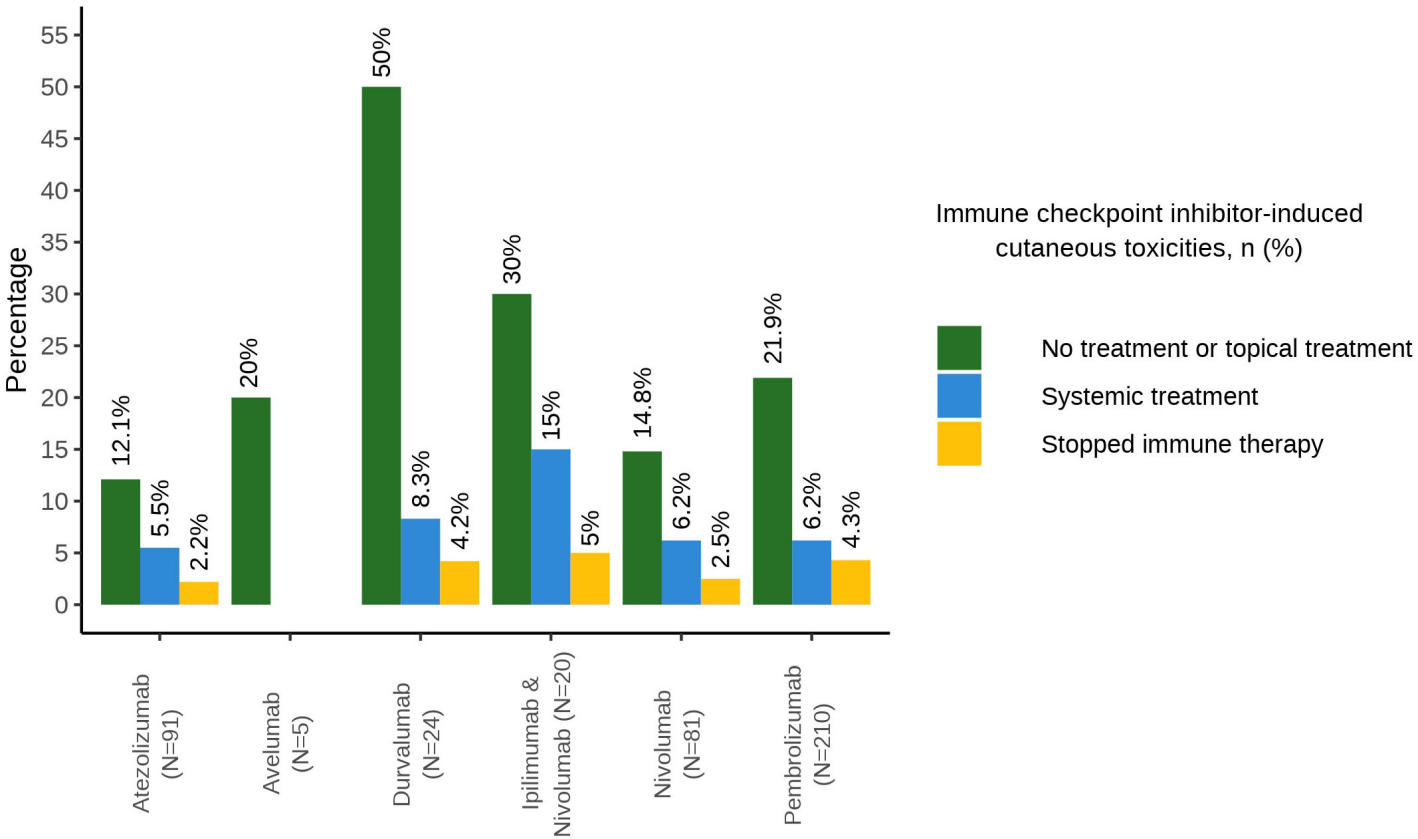
Barplot with treatment decisions of cutaneous adverse events.

**Figure 2** summarizes the treatment decisions and corresponding clinical classifications of severity for each administered immunotherapy. 15.0% of the patients (n=3/20) had to be treated with systemic corticosteroids or antihistamines due to ir-cAEs during treatment with ipilimumab and nivolumab (moderate ir-cAE). One out of the 20 patients who received therapy with ipilimumab and nivolumab had to stop treatment due to severe ir-cAEs. 12 out of 81 patients under nivolumab alone developed mild ir-cAE and required no or only topical therapy (14.8%), five received systemic therapy (6.2%), and 2 had to discontinue therapy due to severe ir-cAEs (2.5%). Ir-cAEs due to the therapy with durvalumab were mostly mild (n=12/24, 50.0% no treatment or topical treatment). Most of the side effects due to the therapy with pembrolizumab and atezolizumab were mild and needed no or only topical therapy (21.9%, n=46/210; 12.1%, n=11/91 respectively). Only few of these patients had to stop the therapy with CPIs because of side effects [4.3% due to pembrolizumab (n=9/210) and two patients due to atezolizumab (2.2%)]. The ir-cAEs caused by avelumab did not require any treatment.

In total 361 patients had a documented reason for discontinuing CPI therapy. Apart from ir-cAE, the most common reasons for early discontinuation of CPI in our study were tumor progression (n=174/361, 48.2%), death (n=49/361, 13.6%) and patient request (n=17/361, 4.7%). These were followed by immune-related adverse events such as colitis (n=16/361, 4.4%), hepatitis (n=16/361, 4.4%) and pneumonitis (n=14/361, 3.9%).

## 4 Discussion

This retrospective study of CPI-induced dermatological side-effects showed that immunotherapies lead to ir-cAE in about one third of the treated patients. Nonetheless, ir-cAE are mostly mild and are self-limiting or require only topical treatment.

This is consistent with the literature, where cutaneous side effects have been reported with a frequency of 30-60% of cases and have also shown a mild and self-limiting course that has not led to discontinuation of treatment (7, 10, 16, 35–42). A minority of patients (6.2%) were treated with systematic therapy (corticoids or antihistamines) and the majority of them exhibited a favourable response, only 2.5% had to discontinue the immunotherapy. However, prior to the discontinuation of immunotherapy due to cutaneous adverse events not responding to systemic corticoids, the administration of biologics remains a viable therapeutic option. Individual studies have demonstrated the efficacy of this approach (43, 44). Nevertheless, the precise impact of biologics on tumour development remains inconclusive. In our study, none of the patients received biologics for the treatment of ircAE.

No life-threatening events like SJS/TEN or DRESS were documented within the 3 years of our study. As the ir-cAEs were mostly mild, it was rarely necessary to refer the patients to a dermatologist (less than 20%, n=24/131).

Nevertheless, close interdisciplinary collaboration between the treating oncologists and dermatologists is important, as even mild side effects may be harbingers of serious adverse events, for example pruritus in (non-bullous) bullous pemphigus (10).

In our study, durvalumab caused the highest incidence of ir-cAEs (62.5%), followed by ipilimumab and nivolumab (50%). This contrasts with the existing literature, which describes a higher incidence of ir-cAEs for ipilimumab/nivolumab than for monotherapies (6, 10, 16, 20, 31, 41, 42).

In our study, this ir-cAE rate for durvalumab is mainly based on an incidence of 54.2% (13/24) for pruritus. However, the Summary of Product Characteristics for Imfinzi^®^ (durvalumab), dated 05-APR-2024 on the EMA website (45), based on a safety database of 4045 patients, reports an incidence rate of pruritus of 11.4%, which is still in line with the 12.4% observed in the PACIFIC Phase 3 study (46, 47). We therefore cannot exclude the possibility that this signal based on a few durvalumab patients in our study is an artifact. We could not find any specific patient attributes or other reasons that explain the higher pruritus rate in our study.

Even if durvalumab caused ir-cAE in 62.5% of the patients taking it, the PD-L1 inhibitors as a group showed a notably lower incidence (28.3%) than the ipilimumab/nivolumab combination therapy (50%), which is again in line with the literature (6, 10, 24).

According to literature, ir-cAEs with durvalumab and the other monotherapies were mainly mild, whereas in those studies ir-cAEs with the combination of ipilimumab and nivolumab were more severe and required more often a systemic therapy or had to be discontinued (20, 27, 48).

The most common ir-cAEs were pruritus, maculopapular exanthema followed by xerosis cutis.

Pruritus was documented in 109 out of 131 patients with ir-cAEs. This means that pruritus alone or in combination with another skin lesion such as xerosis cutis or maculopapular exanthema occurred in 83.2% of patients who developed an ir-cAE. As described in literature, pruritus is common with nivolumab and ipilimumab combination therapy and less common with PD-L1 inhibitors in general (16, 27, 38, 44, 49).

The median time to onset of maculopapular exanthema was shorter, while pruritus showed the shortest (0 days) and longest time (>2 years) to onset at the individual level. Despite skin changes being among the first side effects, they have also been reported to occur after more than a year (8).

Patients with melanoma reported the highest proportion of ir-cAEs during CPI treatment. This coincides with the results of an earlier publication (50). Pruritus, maculopapular exanthema, and especially other skin toxicities such as vitiligo were most common in treated melanoma. Moreover, a positive correlation was demonstrated between the development of vitiligo and a better tumor response and a higher survival rate in patients with melanoma (7, 51–54). Earlier retrospective studies have shown a positive impact on tumor response in patients who developed ir-cAEs due to CPI (7, 55–58).

No baseline characteristics of the patients were clearly distinct between those with and without ir-cAE, which could have partly explained the development of immune-related adverse events. It is still unclear why some patients develop ir-cAE while others do not. Different studies showed a relation to HLA variants. For example, Hasan Ali O. et al. showed the frequency of specific HLA variant was significantly higher in patients suffering from pruritus while receiving immunotherapy (59). This suggests a genetic component and may be a helpful prognostic factor predicting the development of ir-CAE in the future. Finally allergic mechanisms, mainly T-cell mediated type IV hypersensitivities, are likely to be responsible for some ir-cAE. Beside a hypersensitivity to the checkpoint inhibitor substance itself, the latter may induce hypersensitivities to concomitant medications (60, 61).

### 4.1 Limitations

Although we were able to obtain a large amount of data from patients who received immunotherapy, one limitation is its retrospective design. Retrospectively, the data could not be classified according to the CTCAE criteria, and therefore was not recorded in a structured and standardized manner. Reporting bias, especially for mild ir-cAEs, cannot be excluded. In addition, this study is limited to one study center. Consequently, the findings cannot be fully generalized to other regions or countries. Although other reasons for skin lesions were excluded initially, the complex clinical course of oncological patients and their comorbidities make it difficult to rule out any potential impact on the study results. Our data extract did not record whether the immunotherapies were given as monotherapy or, at least temporarily, in combination with chemotherapy or radiotherapy.

### 4.2 Conclusions

Overall, ir-cAEs were reported in about one third of the patients. Generally, ir-cAEs are mild and may well be treated topically. CPIs are and will continue to be of high importance in anti-cancer treatment. Prompt recognition and treatment of any irAEs is crucial and close interdisciplinary collaboration essential. Not only large prospective studies are warranted, but future studies should also investigate factors increasing the risk of developing immune-related adverse events.

## Data Availability

The raw data supporting the conclusions of this article will be made available by the authors, without undue reservation.
