# Supplementary figures and images for: Cutaneous adverse events due to checkpoint inhibitors – a retrospective analysis at a tertiary referral hospital in Switzerland 2019-2022
Source: Front Oncol. 2024 Dec 5;14:1485594. doi: 10.3389/fonc.2024.1485594 (PMC11655322; doi:10.3389/fonc.2024.1485594)

Barplot with percentages of patients for whom at least one ir-cAE is documented (by CPI)


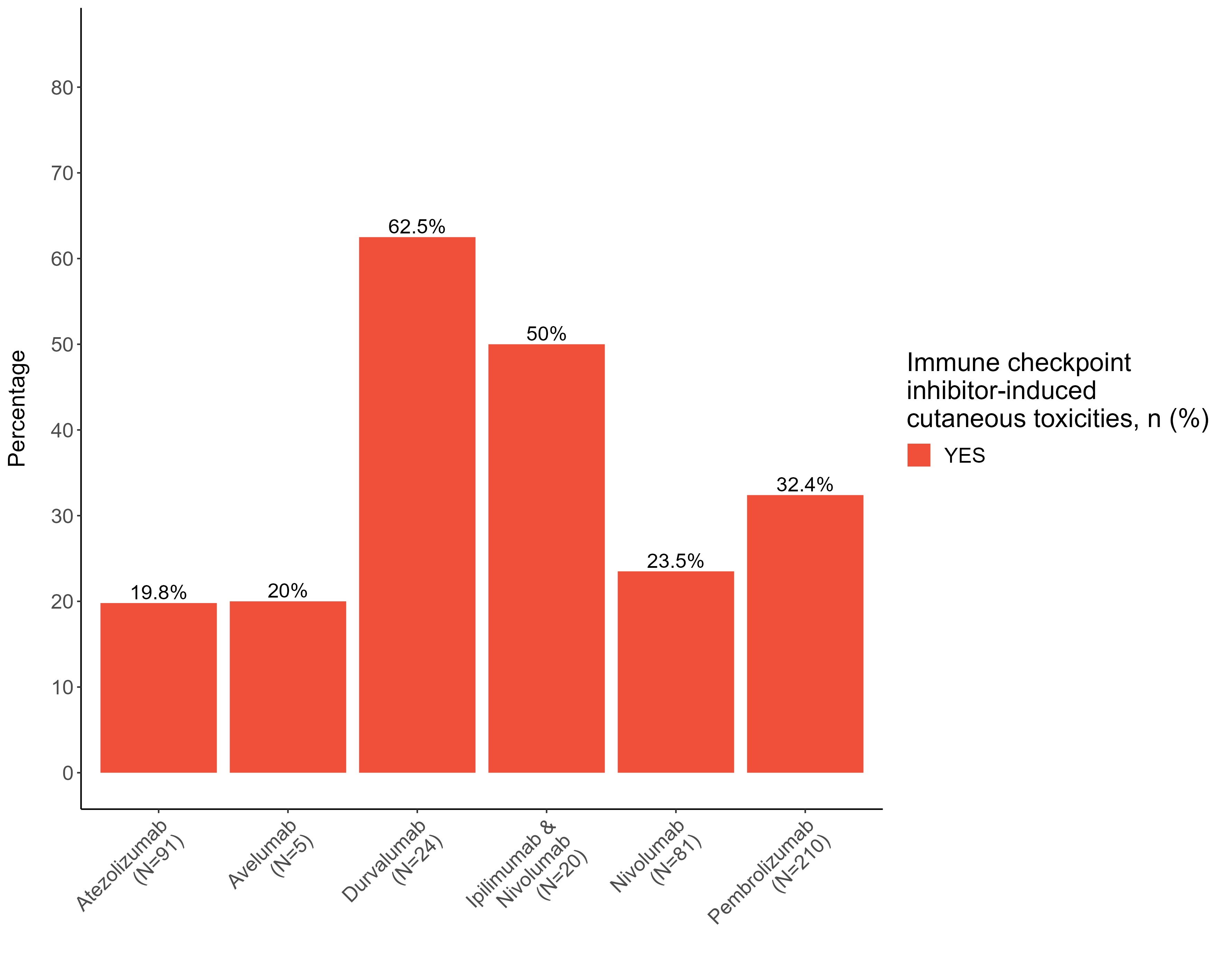

Supplement: Supplementary file 1 [file DataSheet1.docx]
