# Supplementary material for: Cutaneous adverse events due to checkpoint inhibitors – a retrospective analysis at a tertiary referral hospital in Switzerland 2019-2022
Source: Front Oncol. 2024 Dec 5;14:1485594. doi: 10.3389/fonc.2024.1485594 (PMC11655322; doi:10.3389/fonc.2024.1485594)
Supplement: Supplementary file 2 [file Table1.docx]

Supplements

Materials and Methods

To assess the association between cutaneous adverse events and immunotherapy type, we developed exploratory regressions models, presented in the Results section of the Supplement. These include unadjusted and adjusted logistic regression models with “cutaneous adverse event (yes/no)” as the outcome and “immunotherapy type” as the variable of interest. The adjusted model also accounts for the variables “primary cancer”, “age”, and “sex”.

2. Regression table: Unadjusted and adjusted logistic regression models with “cutaneous adverse event (yes/no)” as outcome to assess its association with immunotherapy type, with the adjusted model containing the variables “primary cancer”, “age”, and “sex” for adjustment.

|  | Unadjusted | | | Adjusted | | |
| --- | --- | --- | --- | --- | --- | --- |
| **Characteristic** | OR^a^ | 95% CI^a^ | p-value | OR^a^ | 95% CI^a^ | p-value |
| (Intercept) | 0.48 | 0.36, 0.64 | <0.001 | 0.32 | 0.08, 1.26 | 0.11 |
| **Immunotherapy, n (%)** |  |  |  |  |  |  |
| Pembrolizumab | — | — |  | — | — |  |
| Atezolizumab | 0.51 | 0.28, 0.91 | 0.028 | 0.59 | 0.31, 1.08 | 0.10 |
| Avelumab | 0.52 | 0.03, 3.61 | 0.56 | 0.59 | 0.03, 4.66 | 0.66 |
| Durvalumab | 3.48 | 1.47, 8.67 | 0.005 | 3.21 | 1.30, 8.34 | 0.013 |
| Ipilimumab &    Nivolumab | 2.09 | 0.82, 5.32 | 0.12 | 1.70 | 0.62, 4.67 | 0.30 |
| Nivolumab | 0.64 | 0.35, 1.14 | 0.14 | 0.72 | 0.36, 1.39 | 0.34 |
| **Primary cancer, n (%)** |  |  |  |  |  |  |
| Malignant neoplasms of respiratory and intrathoracic organs (C30-39) |  |  |  | — | — |  |
| Malignant neoplasms of lip, oral cavity and pharynx (C00-14) |  |  |  | 0.99 | 0.37, 2.49 | 0.99 |
| Malignant neoplasms of digestive organs (C15-26) |  |  |  | 0.61 | 0.25, 1.34 | 0.23 |
| Melanoma and other malignant neoplasms of skin (C43-44) |  |  |  | 1.38 | 0.72, 2.62 | 0.33 |
| Malignant neoplasm of breast and female genital organs (C50-C63) |  |  |  | 0.52 | 0.15, 1.49 | 0.25 |
| Malignant neoplasms of urinary tract (C64-68) |  |  |  | 0.79 | 0.34, 1.74 | 0.57 |
| Other malignant neoplasm (<10 patients)^b^ |  |  |  | 0.13 | 0.01, 0.68 | 0.054 |
| **Age [years]** |  |  |  | 1.01 | 0.99, 1.03 | 0.48 |
| **Sex, n (%)** |  |  |  |  |  |  |
| Male |  |  |  | — | — |  |
| Female |  |  |  | 1.10 | 0.68, 1.77 | 0.69 |
| ^a^OR = Odds Ratio, CI = Confidence Interval, ^b^Other neoplasm include 7 patients with sarcoma, 5 patients with neuroendocrine carcinoma, 4 patients with Hodgkin´s lymphoma, 4 patients with prostate carcinoma. | | | | | | |
